# Smartwatch data from non-competitive athletes during the GiroE-2024 e-bike multi-stage race

**DOI:** 10.1016/j.dib.2025.112440

**Published:** 2026-01-03

**Authors:** Paolo Bellagente, Dennis Brandāo, Salvatore Dello Iacono, Paolo Ferrari, Alessandra Flammini, Massimiliano Gaffurini, Luigi Gaioni, Paolo Malighetti, Stefano Rinaldi, Emiliano Sisinni, Matteo Verzeroli

**Affiliations:** a University of Brescia – Information Engineering Department Via Branze 38 25123 Brescia (BS) Italy; b University of Bergamo – Engineering and Applied Science Department Viale Marconi, 5 24044 Dalmine (BG) Italy; c University of Bergamo – Management, Information and Production Engineering Department via Pasubio 7b 24044 Dalmine (BG) Italy; d University of Bergamo – PhD School Viale Papa Giovanni XXIII 106 24121 Bergamo (BG) Italy

**Keywords:** Accelerometers, Heart rate, Cycling tracks, Activity tracking

## Abstract

This article presented the data collected with 9 Garmin Fēnix® 7 - Standard Edition smartwatches during the GiroE-2024. GiroE is an annual e-bike event that runs concurrently with the Giro d'Italia, one of the most prestigious and challenging road cycling races worldwide. Participants ride along selected stages of the Giro d'Italia route, enjoying the same terrains and atmosphere on pedal-assisted bicycles.

The data was collected with an ad-hoc application installed on each smartwatch, purposely developed to log raw data from all available sensors at the maximum sampling frequency possible. The resulting dataset contains raw data from location sensor (based on global navigation satellite system GNSS), heart rate sensor (using photoplethysmography -PPG- and taking advantage from the Garmin Elevate proprietary technology), barometric altimeter, digital compass, tri-axial accelerometer, and thermometer.

The data was collected during the programme ‘Strengthening of research structures and creation of national R&D champions on certain Key Enabling Technologies’ by the ‘National Centre for Sustainable Mobility', Spoke N° 5 - ‘Light Vehicle and Active Mobility’, funded by the Italian Ministry of University and Research with European Union “Next Generation EU” funds.

Therefore, the dataset presented in this article provides raw data supporting research on cyclist behaviour, road conditions, and heart rate modelling. It could be useful in assessing smartwatch data integration and can provide a ground truth for clinical trials made in similar conditions, acting as a “normal” condition for further reference.

Specifications Table

**Table:** 

Subject	Computer Sciences
Specific subject area	Smartwatch use to monitor e-bike non-competitive activity.
Type of data	Comma separated values (CSV) File – Processed cleaned data.
Data collection	Data were manually collected from 9 Garmin Fenix® 7 - Standard Edition smartwatches during the GiroE-2024, a non-competitive e-bike race held in 20 different locations in Italy between the 4th and the 26th of May, 2024
Data source location	**Institution:** Department of Information Engineering, University of Brescia. **City:** Brescia. **Country:** Italy. **Latitude and longitude for collected samples/data:** available in the dataset.
Data accessibility	Repository name: GiroE 2024: Smartwatch data from non-competitive athletes during an e-bike multi-stage race. Data identification number: *DOI*10.5281/zenodo.14882222 Direct URL to data: https://zenodo.org/records/14882223 Instructions for accessing these data: The data are openly accessible and can be downloaded directly from the provided URL. No special permissions or credentials are required for access.

## 1 Value of the Data

• The data refers to the GiroE-2024. Stages and tracks follow the calendar of the Giro d'Italia 2024, the second most important stage race worldwide and are exclusively carried out with pedal-assisted racing bicycles. Only the starting locations and stage mileage change, making data useful in evaluating similar data collections.
• The data could be used as ground truth about physical conditions and performance of e-bike non-agonistic rides.
• The data could be used to assess analysis techniques to estimate road surface roughness, cyclist behaviour and heart rate on different tracks.
• The data could be used as a benchmark to identify which measures must be taken or which dataset can be merged alongside smartwatch data to reach the research goals, avoiding unnecessary costs and efforts.
• The data could be compared with other datasets, including clinical trials, also considering that all the participants have been granted with a “non-competitive sports medical certificate” provided by a sports medicine specialist as required by the Italian law.

## 2 Background

The MOST project is a key initiative funded by the Next Generation EU program under the National Recovery and Resilience Plan. It aims to advance research on sustainable mobility across various domains, including air, road, rail, water transportation, light vehicles, and active means of transport. The project comprises 14 Spokes, each dedicated to a specific area of research; in particular, Spoke 5 focuses on light vehicles and active mobility. The MOST project is investigating the application of advanced technologies in light electric vehicles. This includes the integration of sensor networks, Internet of Things (IoT) devices, and vehicle-to-everything (V2X) infrastructures. The MOST project has established work package WP4.1 to advance key enabling technologies, including sensors, ICT infrastructures, and wearable modules, for gathering reliable data on vehicles, users, and their environment. During the Giro-E, a cycling competition that follows the roads and days of the Giro d'Italia in May 2024, smartwatches were entrusted to cyclists of one team, who wore them during the event. During a 20-day campaign with varying environmental conditions, a range of users with varying cycling backgrounds, from beginners to pros, and tracks with varying degrees of difficulty, this event made field testing and real-world data collection possible.

## 3 Data Description

The dataset contains data recorded by onboard sensors embedded in 9 Garmin Fenix S7 during the GiroE-2024, a 20-stage bicycle touring event exclusively carried out with pedal-assisted racing bicycles (E-BRO 3.0 bikes by Olmo), homologated and hosting 250 W motors (E-P3+ GP Polini motor) and a 500 Wh battery (by Polini) with a maximum speed limited to 25 km/h. The 20 stages globally feature 1100 km of different cyclable terrains. The total recorded distance is 3749.843 km for a total recording length of 180.93 hours of e-bike rides. The dataset consists of a single comma-separated values (CSV) file named giroe2024.csv. It is compressed in a 155.63 MB ZIP archive named giroe2024.csv.zip. Uncompressed, the CSV file size is approximately 455.2 MB, and it includes 625703 rows in 24 columns. Detailed column descriptions are reported in Table 1.

**Table 1:** Dataset columns definitions.

Column	Description
**Device**	Is an integer serial number of the recording smartwatch
**Session**	Is the timestamp of the first sample of a recording used to identify a recording itself [datetime]
**timestamp**	Is the timestamp of the sample [datetime]
**position_lat**	Is the latitude of the sample in decimal degrees, gathered using the Garmin IQ function Position.enableLocationEvents, with LOCATION_CONTINUOUS and POSITIONING_MODE_NORMAL options.
**position_long**	Is the longitude of the sample in decimal degrees, gathered using the Garmin IQ function Position.enableLocationEvents, with LOCATION_CONTINUOUS and POSITIONING_MODE_NORMAL options.
**Distance**	Is the distance traveled from the start of the session in kilometers [km]
**heart_rate**	Is the athlete hearth rate in beats per minute, gathered by the smartwatch os interface [bpm]
**SystemBattery**	Is the smartwatch battery percentage [%]
**PositionLatitude**	Is the latitude of the sample in decimal degrees, gathered using the Garmin IQ function Position.enableLocationEvents, with LOCATION_CONTINUOUS and POSITIONING_MODE_NORMAL options.
**PositionLongitude**	Is the longitude of the sample in decimal degrees, gathered using the Garmin IQ function Position.enableLocationEvents, with LOCATION_CONTINUOUS and POSITIONING_MODE_NORMAL options.
**PositionAltitude**	Is the altitude in meters over the sea level, gathered using the Garmin IQ function Position.enableLocationEvents, with LOCATION_CONTINUOUS and POSITIONING_MODE_NORMAL options. [m]
**PositionSpeed**	Speed in meters per second from the GNSS sensor interface [m/s] using the Garmin IQ function Position.enableLocationEvents, with LOCATION_CONTINUOUS and POSITIONING_MODE_NORMAL options. Speed is derived from the most accurate source in the following order: 1. GNSS 2. Accelerometer
**PositionHeading**	Heading in radiants with respect to the true north, using the Garmin IQ function Position.enableLocationEvents, with LOCATION_CONTINUOUS and POSITIONING_MODE_NORMAL options. [rad]
**PositionAccuracy**	Enumeration with indication of Position Quality: 0 – Not available 1 – last known 2 – poor 3 – usable 4 – good using the Garmin IQ function Position.enableLocationEvents, with LOCATION_CONTINUOUS and POSITIONING_MODE_NORMAL options.
**SensorAltitude**	Is the barometric or GNSS altitude in meters over the sea level, gathered using the Garmin IQ function Sensor.SetEnableSensors with suitable callbacks. If GNSS is not available then barometer altitude readings are used [Pa].
**SensorSpeed**	Speed in meters per second gathered using the Garmin IQ function Sensor.SetEnableSensors with suitable callbacks. [m/s] Speed is derived from the most accurate source in the following order: GNSS Accelerometers
**SensorHeading**	Heading in radiants with respect to the true north, gathered using the Garmin IQ function Sensor.SetEnableSensors with suitable callbacks. [rad]
**SensorPressure**	The barometric pressure in Pascals [Pa].
**SensorAccelerationX_HD**	An Array of 25 values of acceleration over the x-axis, expressed millig-units, gathered using the Garmin IQ function Sensor.SetEnableSensors with suitable callbacks [$g\cdot 10^{-3}$]
**SensorAccelerationY_HD**	An Array of 25 values of acceleration over the y-axis, expressed millig-units, gathered using the Garmin IQ function Sensor.SetEnableSensors with suitable callbacks. [$g\cdot 10^{-3}$]
**SensorAccelerationZ_HD**	An Array of 25 values of acceleration over the z-axis, expressed millig-units, gathered using the Garmin IQ function Sensor.SetEnableSensors with suitable callbacks. [$g\cdot 10^{-3}$]
**SensorHeartrate**	Is the athlete hearth rate in beats per minute, gathered using the Garmin IQ function Sensor.SetEnableSensors with suitable callbacks. [bpm]
**SensorTemperature**	The temperature in degrees Celsius gathered using the Garmin IQ function Sensor.SetEnableSensors with suitable callbacks. [°C]
**Date**	Date of the recorded session

Each sensor is sampled by the smartwatch every second (1 Hz) except for accelerometer axes that are sampled every 40 ms (25 Hz).

Data is available for each of the 20 GiroE-2024 stages, starting from 04/05/2024 to 26/05/2024, but due to the willingness of participants, which could change stage by stage, the number of recorded devices can vary. A summary of the collected data by each device, stage by stage, is reported in Table 2.

**Table 2:** Collected data summary.

Stage Device	Total Recording Time [min]																			
	1	2	3	4	5	6	7	8	9	10	11	12	13	14	15	16	17	18	19	20
4493	100	0	170	0	0	0	0	0	0	0	143	189	0	110	0	145	0	195	0	0
4526	148	0	167	0	180	0	0	0	0	260	0	0	0	13	0	145	0	195	0	90
4595	89	0	0	0	0	0	0	0	0	0	143	0	0	0	240	145	0	195	0	0
3818	0	73	0	255	0	171	0	0	133	0	0	0	0	0	240	0	215	0	0	90
4141	0	75	0	255	0	0	0	182	0	0	0	0	150	0	240	0	215	195	0	90
4253	0	75	0	255	0	171	0	195	0	260	0	190	0	110	0	145	0	195	166	90
4530	0	75	0	255	0	0	86	0	133	0	0	0	0	0	240	0	215	195	0	90
4642	0	75	0	0	0	0	0	0	0	0	143	0	150	110	192	0	164	0	166	79
4155	0	0	180	0	0	0	0	0	133	0	143	0	126	0	240	0	0	0	166	0
Number of devices	**3**	**5**	**3**	**4**	**1**	**2**	**1**	**2**	**3**	**2**	**4**	**2**	**3**	**4**	**6**	**4**	**4**	**6**	**3**	**6**

The devices recorded per stage varies from 1 to 6 due to the changes in the number of team member and their availability to participate to the data collection. The duration of recordings varies as each stage has a different length. In some cases, due to the track difficulty, some participants could have withdrawn before the end of the stage. As an example, in the first stage the athlete wearing the device nr. 4595 withdrawn from the race after 89 minutes. It must be reminded that the participation to the data collection is on voluntary base and as an example at the stage 7 only one participant decided to take part to the experimentation.

**Fig. 1:**
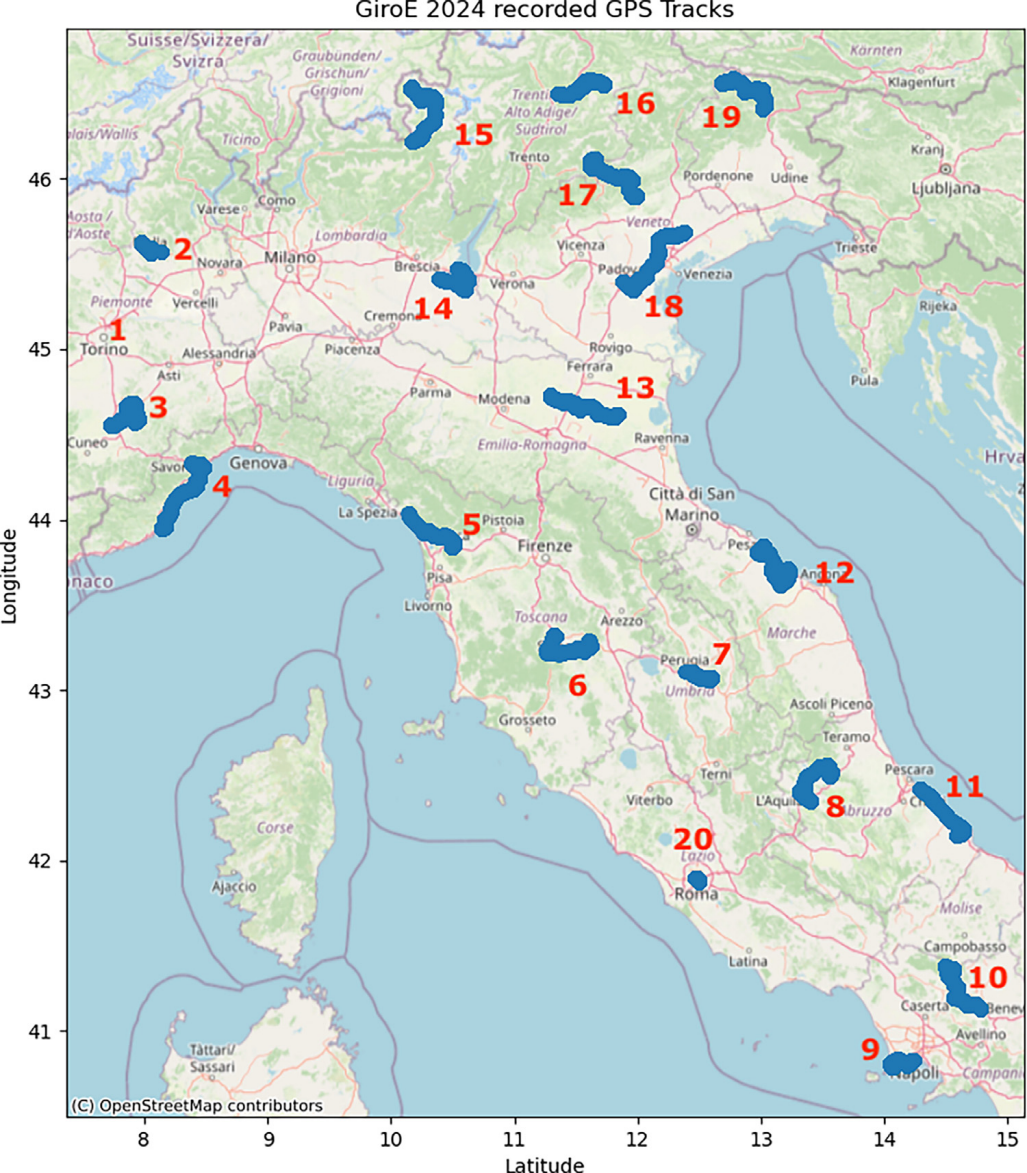
Geographical locations of the 20 stages of the GiroE-2024.

Fig. 1 shows the geographical locations of the 20 stages of the GiroE-2024. It should be noted that the first stage in Turin does not have GNSS data because the bikers inadvertently stop the application (hereinafter the “MOST Garmin Raw Logger” app) from logging these data simply pressing one of the smartwatch buttons. In order to avoid similar issues, this feature was enabled by a specific button sequence since stage 2. Although the sensor interface data is missing, stage 1 recording has not been removed, as the other data of interest (e.g., heart rate, distance…, etc.) are present and still useful for some analysis.

**Fig. 2:**
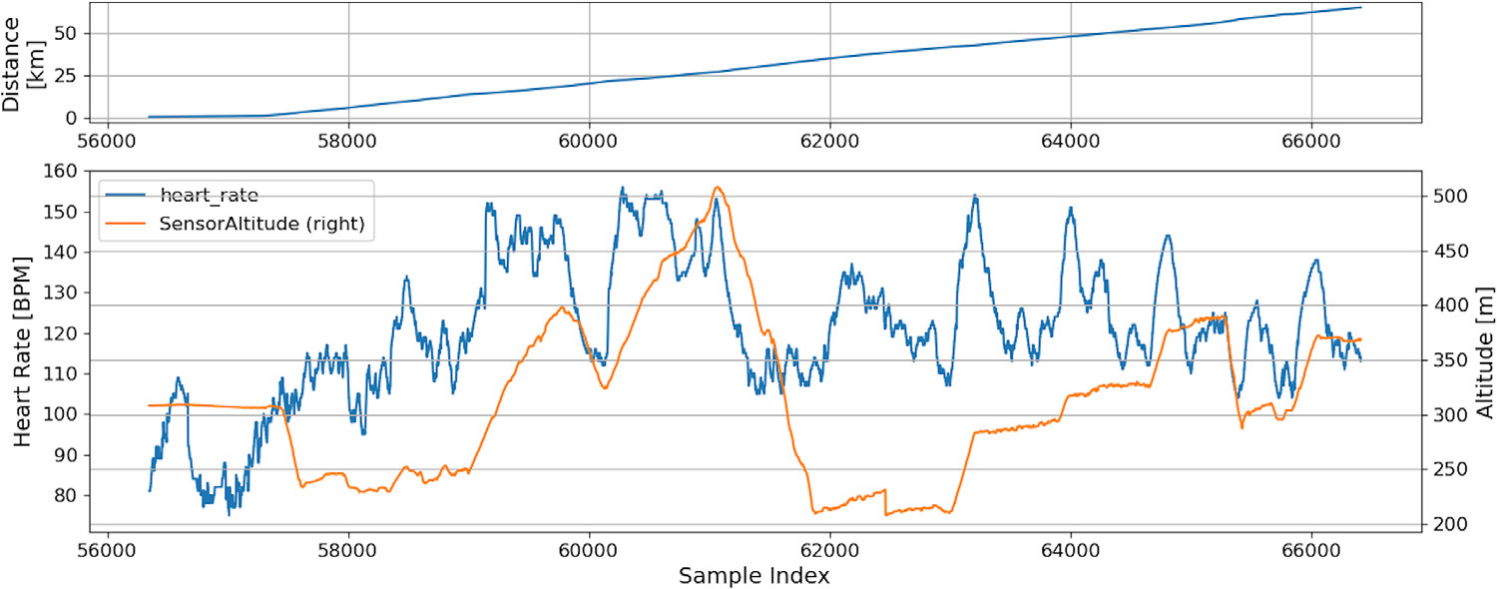
Example of performance data.

An example of performance data is shown in Fig. 2, where heart rate, distance and altitude have been compared for a single device in a single stage. As it can be seen in this specific graph, this dataset permits to highlight many peculiarities caused by the use of e-bikes, in fact the heartrate could decrease not only due to the changes in the road slope but also due to the use of the electric motor support, that is under the cyclist control.

## 4 Experimental Design, Materials and Methods

**Fig. 3:**
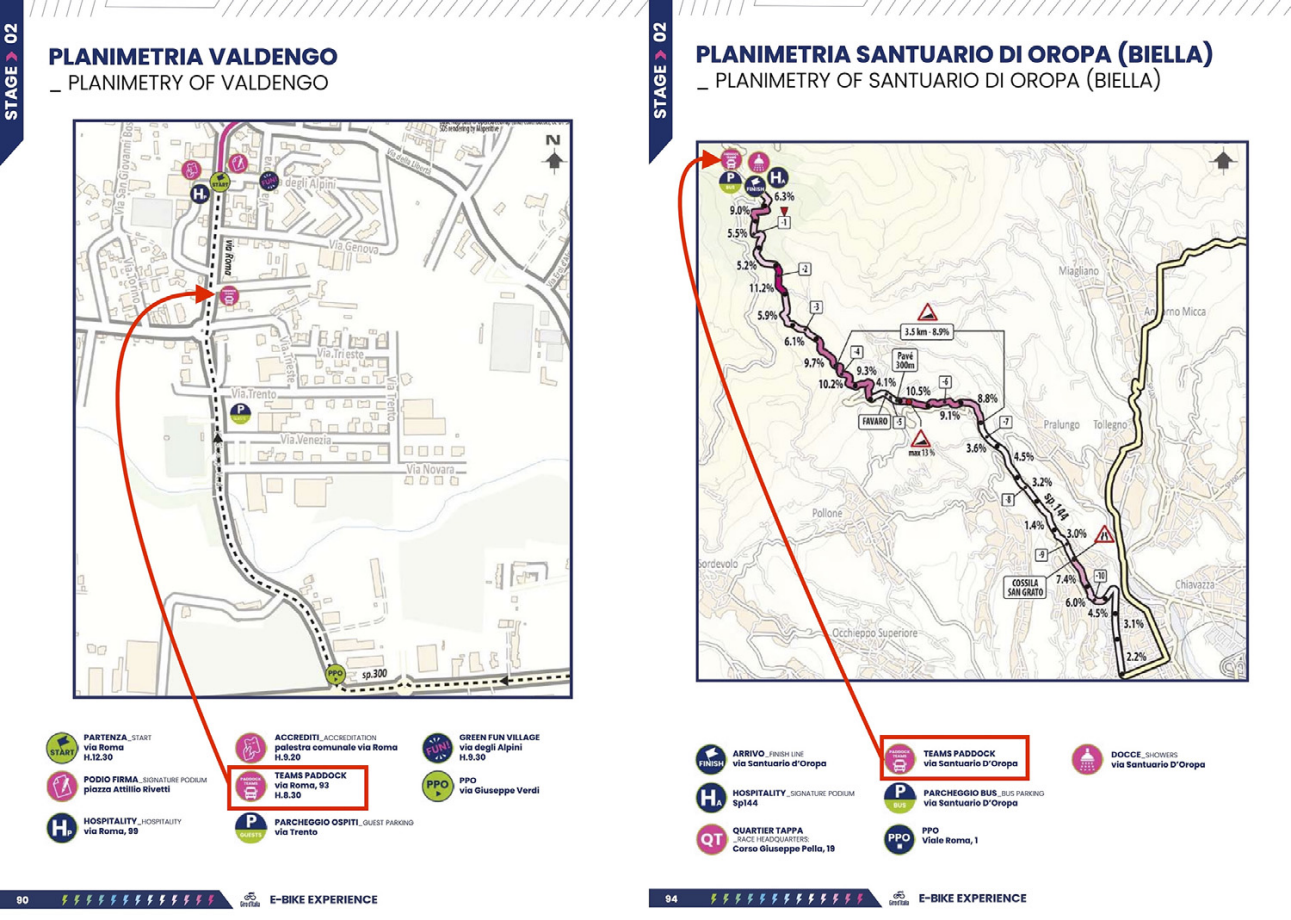
Extract of the race roadbook for Stage 2 05/05/2024.

The first step of the experimental design was to understand the daily routine of the GiroE while identifying the best procedure to interact with athletes without interfering too much with the race routine. Fig. 3 depicts an example of the logistic organisation of the event. The left page of Fig. 3 shows the Stage 2 Start Planimetry, while the right page shows the Stage 2 Arrival Planimetry. A Team Paddock was planned in both Start and Arrival locations [1].

The participant management of the considered event is different with respect to a classic bike race. Indeed, regularly registered teams, depending on the individual commercial agreements, can be classified in the following way [2]:

• **Official team:** This is the Team which participates in all the stages of the Event and competes in the allocation of the classification jerseys;
• **Daily team:** This is the team which participates in one or more stages but does not compete for the allocation of the classification jerseys.

**Fig. 4:**
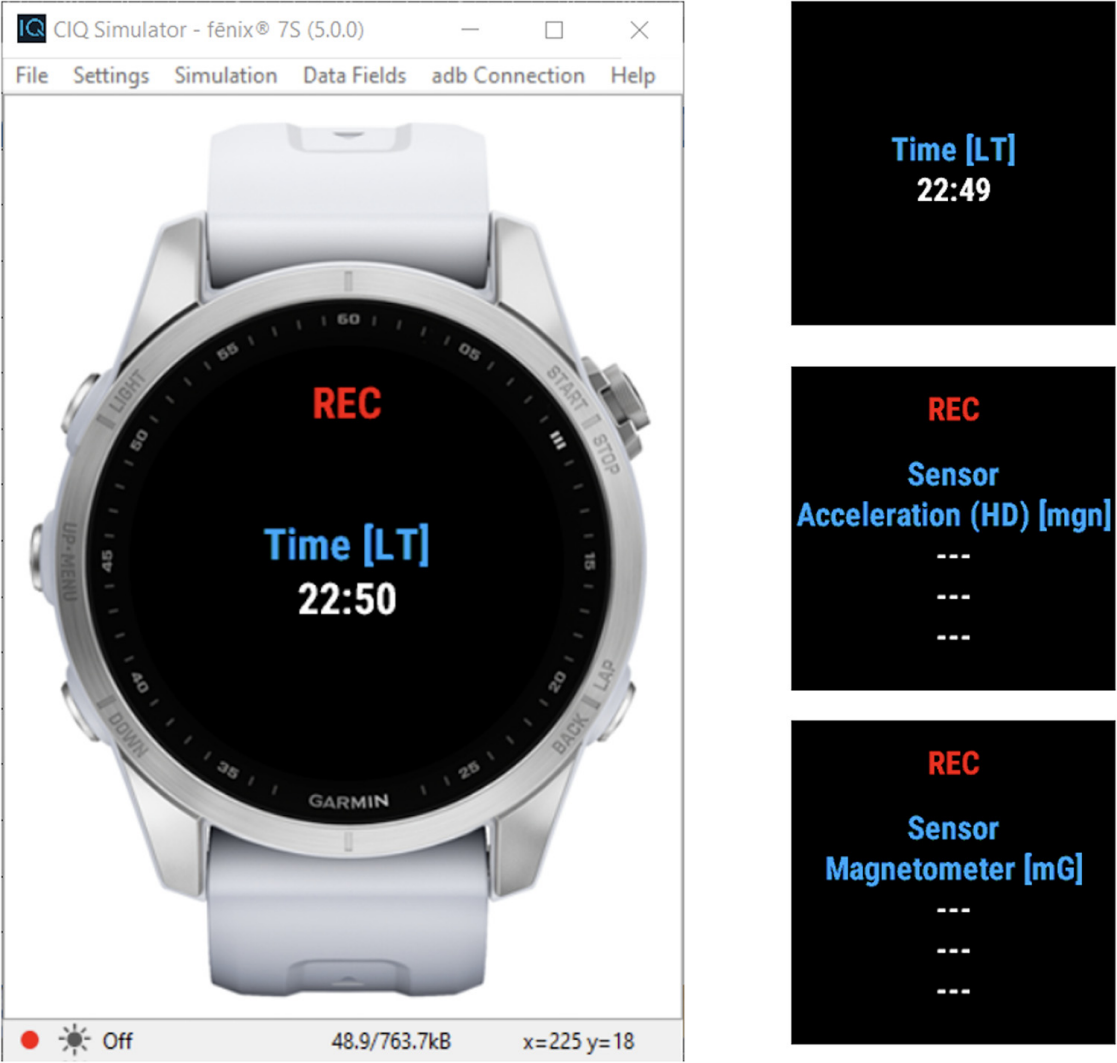
A screenshot of the developed smartwatch app MOST garmin raw logger.

Paddocks were used by teams to deliver the branded e-bikes to athletes and to make the necessary mechanical setup. Every day, at a team’s suitable time, a researcher showed up at the start paddock to:

• Collect athletes’ signatures to privacy consensus as athletes’ availability can change stage by stage;
• Deliver the smartwatch to collect data, gaining a receipt signature from the athlete;
• Ensure the athlete starts the MOST Garmin Raw Logger app Fig. 4 to collect data.

At the end of the race, a researcher showed up at the arrival paddock to:

• Collect delivered smartwatches from athletes;
• Download the collected data and upload it to a cloud repository as a backup.

Only one of the participating teams established an agreement with the research team to participate to the data collection. Both official team and daily team members were allowed to take part to the dataset collection, however, the participation was on athletes’ voluntary basis that could be different at each stage. In any case, any athlete underwent an evaluation by a sports medicine specialist, who issued the required certificate according to Italian law to assess the minimum health condition [4]. The smartwatch was provided by the research team to each cyclist on daily basis.

The smartwatch identified as suitable for the experiments was the Garmin Fenix 7 - Standard Edition. It is a premium multisport smartwatch designed for outdoor enthusiasts, athletes, and adventurers. It represented one of the commercial reference smartwatches for bike activity, according to non-competitive athletes and bike enthusiasts consulted.

To create a reliable and efficient tool for recording and analysing smartwatch sensor data in real time, the aforementioned “MOST Raw Logger” app has been developed [3], leveraging the Garmin IQ Framework, and its interface is shown in Fig. 4. In particular, this application is targeted to the Garmin Fenix 7 smartwatches and, even being compatible with different devices, it has been tested only on this specific model.

The application provides users with an easy-to-use yet powerful data-logging solution. Unlike traditional activity tracking applications that process and filter sensor data before displaying it, MOST Garmin Raw Logger ensures that all raw data is captured without modification. This unprocessed data is particularly valuable for developers, researchers, and athletes who require precise measurements for in-depth analysis.

The application has been developed to stand the difficulties of the racing event and allow the researcher to obtain the maximum amount of recording independently from the cyclist’s actions and settings. The application requires only to be opened to start recording. To stop recording, the app requires a specific button sequence in order to eliminate involuntary logging losses from the athletes, as observable in stage 1 recorded data. All sensors are acquired using the maximum sampling rate available for the running device, and measured data is stored in FIT files. Files are intended to be later analysed using Garmin tools like the FIT SDK. The application is fully compatible with the Garmin Connect cloud. However, some difficulties were encountered while automatically syncing the device with the cloud using the standard methods for smartwatches, such as wireless or Bluetooth connection. Log sessions are indeed very long as they were started before the beginning of the race and sometimes terminated hours later due to the availability of athletes. In addition, due to the itinerant nature of the event, a lack of a stable internet connection was often experienced. Such conditions continuously trigger the Garmin Cloud timeouts. For these reasons, the app has been designed to permanently store FIT files on the device and retrieve them using a USB connection, as the device is detected as a storage peripheral when connected to a computer with its proprietary cable. No automation has been provided to extract FIT files, which were manually collected at the end of the event from every single device. Once collected, FIT files are initially converted to JSON structures and independently saved by using the “fitjson” python module available within the python library “fitdecode” [5]. The obtained JSON data files were parsed, and the extracted measure values were saved in CSV files. Then, each file was examined, searching for inconsistent recordings as traces recorded during the pre-race warm-up or the post-race cool-down periods. To clean the dataset, the expected starting and arrival times of each stage were extracted from the Race Roadbook [1] to roughly trim each trace in a timeframe centred around the race stage.

Eventual traces of athletes who interrupted the tracking have been included in the dataset as such traces could contain useful information

## Limitations

The main limitation of this study is the small number of traces available, as only one team agreed to participate in the data collection. Furthermore, the number of devices worn was not constant across the various stages because team composition changed at each stage and athletes’ willingness to participate varied: some athletes chose not to use the smartwatches, while others felt uncomfortable with the idea of being monitored. In addition, despite the recommendations provided by the research team during the devices’ handover, the correct positioning of the smartwatches could only be checked before the start of each stage when athletes were wearing them. Position changes may therefore have occurred during the race, depending on athletes’ preferences and wearing comfort. Finally, due to the limited sample size, tracking individual athletes across stages was avoided to prevent any potential risk of identity disclosure, which made it impossible to analyse behaviour across the entire tour.

## Ethics Statement

The authors confirm that relevant, informed consent was obtained from the athletes who wore the smartwatches. As the experimentation was not medical research but data collection normally made during fitness activities, the Declaration of Helsinki and the Ethical Committee approval are not applicable.

## Credit Author Statement

**Paolo Bellagente:** Methodology, Software, Data Curation, Investigation; **Dennis Brandāo:** Investigation, Writing Review & Editing; **Salvatore Dello Iacono:** Methodology, Software, Investigation, Review & Editing; **Paolo Ferrari:** Investigation, Writing Original Draft; **Alessandra Flammini:** Conceptualisation, Investigation, Supervision, Funding Acquisition; **Massimiliano Gaffurini:** Software, Investigation**; Luigi Gaioni:** Investigation, Writing Review & Editing; **Paolo Malighetti:** Investigation, Project Administration; **Stefano Rinaldi:** Investigation, Writing Review & Editing; **Emiliano Sisinni:** Investigation, Formal Analysis, Review & Editing; **Matteo Verzeroli:** Investigation.

## Data Availability

ZoteroGiroE 2024: Smartwatch data from non-competitive athletes during an e-bike multi-stage race (Original data).
